# The right hemicolon fascial system: a narrative review

**DOI:** 10.3389/fsurg.2026.1854867

**Published:** 2026-05-11

**Authors:** Zhiping Huang, Xumu Shen, Ping Chen, Wei Zhang

**Affiliations:** Department of Gastroenterology, Chongqing University Three Gorges Hospital, Wanzhou, Chongqing, China

**Keywords:** CME, Fredet fascia, Gerota fascia, surgical plane, Toldt fascia

## Abstract

Colorectal surgery has entered the era of membrane anatomy. Total mesorectal excision (TME) and complete mesocolic excision (CME) can significantly improve the 5-year survival rate of patients with colorectal cancer. The complete excision of the mesentery is of great significance for the prognosis of patients with colorectal cancer, and the radical evaluation of colorectal cancer is based on the integrity of the mesentery of the resected gross specimen. However, in right colon cancer surgery, there are still significant controversies and uncertainties in the understanding of the abdominal fascial system and surgical plane, such as Toldt's fascia and Fredet's fascia. This situation is further aggravated by the confusion in nomenclature. Since the same fascia often has different terminologies, which hinders academic communication, Since the same fascia often has different terminologies, which hinders academic communication, we conducted a narrative review of the right colon fascia system based on our personal experience and a literature review. We discovered that although the current terminology remains non-uniform, the concept of fusion space seems to be the most useful in surgical practice and has histological justification.

## Introduction

Colorectal cancer ranks as the third most prevalent malignancy and the second leading cause of cancer - related deaths globally. In 2020, there were approximately 1.88 million new cases and nearly 920,000 deaths (1). The advancement of surgical techniques has facilitated the development of surgery based on membrane anatomy (MA). Surgical procedures have entered the era of membrane anatomy, in which operating within avascular natural spaces guided by MA principles has become a characteristic feature of surgical excellence. The conceptual transformation of colorectal cancer surgery commenced with the Total Mesorectal Excision (TME) surgery proposed by Heald et al. in 1982, which was of significant importance in reducing the local recurrence rate and preserving autonomic nerve function (2). Subsequently, in 2009, German scholar Hohenberger et al. put forward the Complete Mesocolic Excision (CME) surgery, suggesting that CME could decrease the postoperative local recurrence rate of colon cancer and enhance the 5-year survival rate (3). A systematic understanding of the anatomical structure of the right hemicolon system is a prerequisite for accurately identifying the surgical plane, achieving precise and sharp separation, and ensuring the operation of instruments along the correct gap (4). Clinical practice in colorectal surgery has demonstrated that the complete resection of the mesentery is of great significance for the prognosis of patients with colorectal cancer, and the radical evaluation of colorectal cancer is based on the integrity of the mesentery of the resected gross specimen (3, 5, 6). However, controversy still persists regarding the right abdominal fascia system in right colon cancer surgery. The primary focus is on the terminological and embryologic inconsistencies within the retrocolic fascial planes of the right colon.

In light of this, The primary objective of this article is to conduct a narrative review of the current anatomical interpretation of the right hemicolon fascial system and to present the author's corresponding understanding.

### Intestinal rotation and the continuity of the mesentery

The fundamental structural components of the mesentery consist of mesothelial cells, a network of connective tissue, and a group of adipocytes distributed within the interstices of the lattice. The mesentery is highly organized and is composed of fat cells of various sizes and quantities, which are tightly packed between fibrous septa (7). The gut tube and its derivatives are suspended from the body wall by mesenteries formed by a double - layered peritoneum that encloses an organ. The intermediate layer of the mesentery serves as a conduit for blood vessels (arteries and veins), lymphatics (ducts and nodes), and nerves to reach the intestine (8, 9).

During the embryonic stage, the midgut rotates 270 degrees counter - clockwise around the superior mesenteric artery as the axis. After the rotation, the right hemi - mesocolon and the small mesentery are separated on either side of the superior mesenteric artery (SMA), and the ileocolic artery, the terminal branch of the SMA, is directed towards the ileocecal region. The cecum and the dorsal part of the ascending colon are attached to the right posterior abdominal wall of the body cavity, and the hepatic flexure of the colon is attached to the pancreaticoduodenal region.

Classical anatomical knowledge suggests that the mesentery of the small intestine, transverse mesocolon, and sigmoid colon are consistently present in adults, while the right and left hemicolon mesocolons can only be identified in a small number of adults, which implies that the mesentery is fragmented (10–12). However, with the application of laparoscopic and robotic surgery, surgical anatomy has become more significant. As a result, it has been confirmed that the mesocolon is, in fact, continuous from the small intestinal mesenteric to the mesorectal level (13–16).

### Visceral peritoneum vs. visceral fascia and parietal peritoneum vs. parietal fascia

During embryonic development, the lateral plate mesoderm on both sides differentiates into two distinct layers. One layer is contiguous with the extra - embryonic mesoderm that envelops the amnion, and it is designated as the parietal mesoderm. The other layer is attached to the extra - embryonic mesoderm covering the yolk sac and is called the visceral mesoderm. These two layers together enclose a newly formed cavity, specifically the intra-embryonic body cavity. The visceral layer of the lateral plate mesoderm, in combination with the embryonic endoderm, constructs the intestinal wall. The parietal mesodermal cells surrounding the intra - embryonic body cavity give rise to a thin membrane, known as the mesothelial membrane, which secretes serous fluid.

There is a fundamental distinction between membranes and fascia. Coffey et al. performed a full - thickness biopsy of all mesentery regions, spanning from the jejunal flexure of the duodenum to the anorectal canal. They found that the mesentery of the small intestine and the free part of the mesentery of the sigmoid colon have medial and lateral surfaces, both of which are covered by the mesothelium. The transverse mesocolon has upper and lower sides, which are also covered by the mesothelium. The left and right mesocolon also have upper and lower surfaces, with the lower surface attached to the Toldt fascia, and all these surfaces are covered by the mesothelium. They are true membranes (17). Some researchers assert that fascia can be used to describe connective tissue and even any membrane - like tissue that can be stratified in the human body (18). Others claim that fascia is a sheath, a sheet, and any aggregate of anatomic connective tissue (19). However, in any case, the fascia is not lined with epithelial cells like the peritoneum; that is, it is not a true “membrane”.

The mesenterium has a smooth surface and is composed of a single layer of cuboidal cells. It shows a remarkable similarity to the mesopleura and also to the peritoneum of the abdominal cavity. The parietal peritoneum lines the medial side of the abdominal wall, while the visceral peritoneum covers the abdominal organs, including the mesentery. Therefore, the mesenchyme covering both sides of the mesentery should be classified as the “visceral peritoneum”. The mesothelium overlying the retroperitoneum should be classified as the “parietal peritoneum”. Hence, it is debatable whether it is appropriate to refer to the mesothelium at the above two positions as “visceral fascia” and “parietal fascia” (20–25).

### Toldt fascia, Fredet fascia, and fusion fascia

The fusion fascia is a thin membranous structure with well-defined boundaries. In terms of its concept, Hayes et al. proposed that when two layers of mesothelial cells are arranged in close proximity, all mesothelial cells disappear, leaving a connective tissue layer (26). By definition, the inside of fusion fascia cannot be dissected. The most typical example of the fusion fascia is Toldt fascia, which is formed by the fusion of the visceral peritoneum of the mesentery and the parietal peritoneum of the posterior peritoneum (11). However, Culligan et al. (13, 17) have different viewpoints. They believe that the mesothelium still exists in the fused visceral peritoneum, and the fused fascia is a fusion space, which is a loose and extensible avascular area. This view is further supported by the study of Liang et al., who suggested that colonic surgery should be performed between the Toldt fascia, which originates from mesenchymal cells, and the peritoneum from mesothelial cells (27). Toldt fascia plays a vital role in colorectal surgery. It provides a highly significant anatomical guide for the operation. If this anatomical pathway is not followed, the ureter, genital vessels, and duodenum may be damaged when entering the retroperitoneum. Alternatively, if it enters the mesentery, it may lead to mesentery injury and severe hemorrhage.

However, in a recent study, Wedel et al. (28) put forward the proposal that eponyms, like Toldt fascia, should not be used because they are considered the root cause of the present controversies, confusions, and communication barriers. In his study, it can be seen that he renamed the Toldt fascia as the parietal peritoneal fascia, which is a membrane-like structure. A careful inspection shows that what he refers to as the parietal peritoneum fascia (Toldt fascia) is actually the parietal peritoneum of the retroperitoneum. However, the surgical operation plane mentioned in the article, which is the plane between the parietal peritoneal fascia and the mesocolic fascia, is actually the Toldt space. Moreover, the loose connective tissue within it is the Toldt fascia as considered by Coffey et al. Thus, the cause of the confusion is that the Toldt's fascia they refer to is not the same fascia.

The Fredet fascia was initially described by Fredet as the plane of adhesion between visceral peritoneum of the mesocolon of ascending colon and hepatic colonic fexure and the visceral peritoneum of duodenum and pancreas. Garcia-Granero et al. (29) proposed that it serves as an important embryological landmark during right hemicolectomy, which can reduce the incidence of intraoperative complications. Nevertheless, the understanding of the Fredet fascia remains controversial or ambiguous. According to Mike et al. (30, 31), the right Toldt fascia bifurcates into the ventral Fredet fascia and the dorsal Treitz fascia near the descending duodenum, and the Fredet fascia is a type of fusion fascia that cannot be dissected. Consistent with this view, in Wedel's study (28), it can be observed that the Fredet fascia indicated in the article is the prepancreaticoduodenal fascia. However, in the study of Garcia-Granero et al. (29), it can be noted that the Fredet fascia is more likely to be loose connective tissue and is the adhesion plane between the visceral peritoneum of the mesocolon and the visceral peritoneum of the pancreaticoduodenum, rather than a fusion fascia, and the surgical operation is performed in the Fredet fusion space. Once again, the reason for this confusion is that the Fredet fascia they refer to is not the same fascia either. Table 1 presents the understanding of the right hemicolon fascial system by various researchers.

**Table 1:** Different elaborations on the right hemicolon fascial system.

Research	Eponym	Embryologic interpretation	Postnatal anatomy	Surgical plane
Wedel et al. (28)	Toldt fascia	Parietal peritoneum	Parietal peritoneal fascia	1. Plane between the Toldt fascia and the mesocolic fascia
				2. Plane between the Toldt fascia and the anterior renal fascia
	Fredet fascia	Anterior mesoduodenum/pancreas	Pre-duodenopancreatic fascia	Plane between the mesocolon fascia and the Fredet fascia
Coffey et al. (17)	Toldt fascia	Connective tissue layer (Located between the mesothelium of the dorsal mesocolon and the mesothelium of the retroperitoneum)	Connective tissue layer (Located between the mesothelium of the dorsal mesocolon and the mesothelium of the retroperitoneum)	1. Plane between the Toldt fascia and the visceral peritoneum of the mesocolon
				2. Plane between the Toldt fascia and the underlying retroperitoneum
Liang et al. (27)	Toldt fascia	Connective tissue layer (Located between the mesothelium of the dorsal mesocolon and the mesothelium of the retroperitoneum)	Connective tissue layer (Located between the mesothelium of the dorsal mesocolon and the mesothelium of the retroperitoneum)	Plane within the Toldt fascia
Mike et al. (30)	Toldt fascia	Mesothelium of the dorsal mesocolon fused with the mesothelium of the retroperitoneum (all mesothelial cells disappear)	Fusion fascia (Parietal peritoneal fascia)	Plane between the Toldt fascia and deep subperitoneal fascia (anterior renal fascia)
	Fredet fascia	Mesothelium of the dorsal mesocolon fused with the mesothelium of the retroperitoneum (all mesothelial cells disappear)	Fusion fascia (Anterior pancreatic fascia)	Plane between the Pre-duodenopancreatic fascia and the surface of the duodenum/pancreas
Garcia–Granero et al. (29)	Toldt fascia	Connective tissue layer (Located between the mesothelium of the dorsal mesocolon and the mesothelium of the retroperitoneum)	Connective tissue layer (Located between the mesothelium of the dorsal mesocolon and the mesothelium of the retroperitoneum)	Plane within the Toldt fascia
	Fredet fascia	Connective tissue layer (Located between the mesothelium of the dorsal mesocolon and the Mesoduodenum/pancreas)	Connective tissue layer (Located between the mesothelium of the dorsal mesocolon and the Mesoduodenum/pancreas)	Plane within the Fredet fascia

### Anterior and posterior renal fascia or urogenital fascia

After a decade of ambiguity, it has finally become clear that the term “Gerota fascia” should refer to the prerenal fascia (32). In fact, the understanding of the urogenital fascia has been gradually improved. In 1935, Hinman (33) reported that the anterior and posterior fasciae of the kidney and its ureter formed a similar “sandwich” structure. In 1946, Tobin (34) categorized the retroperitoneal fascia into three layers. The middle layer encloses the kidney, ureter, and surrounding connective tissue, and the fibrous connective - tissue fascia formed on its surface is the perirenal fascia, including the Gerota fascia (anterior fascia) and the Zuckerkandl fascia (posterior fascia). Woodburne named it the periureteric fascia in 1977 (33). In 1999, Muntean et al. (35), through autopsy, proposed that the urogenital fascia ended in front of the presacral fascia of the first sacral vertebra and fused laterally with the presacral fascia. In 2014, Yang et al. (36) recognized this layer as the genitourinary hypogastric sheath surrounding the posterior part of the rectum. For right hemicolon surgery, a good understanding of the retroperitoneal fascia system helps in identifying the correct surgical level. If the surgical approach is too deep, it may harm the ureter, genital vessels, hypogastric nerve, etc. If it is too shallow, it may damage the mesocolon.

### Surgical plane involved in the right hemicolon surgery

After gaining an understanding of the right hemicolon fascial system, it is relatively easy to comprehend the operative plane involved in the surgery. Table 1 presents the comprehension of various surgical planes by different investigators. Culligen et al. (17) proposed that most colorectal surgeons disrupt the avascular plane formed between the contiguous mesocolon and fascia (i.e., mesofascial separation), while others disrupt the plane formed by the fascia and the underlying retroperitoneum (i.e., retrofascial separation). Wedel et al. (28) indicated that there are two distinct retrocolic interfaces that can be utilized for the surgical mobilization of the mesocolon: (a) the parieto–mesocolic interface between the parietal peritoneal fascia and the mesocolic fascia, and (b) the parieto–renal interface between the parietal peritoneal fascia and the anterior renal fascia. They regarded the Toldt fascia as a parietal peritoneal fascia, a fusion fascia, and considered it impossible to be dissected. Thus, the surgical plane they mentioned is fundamentally different from the one referred to by Culligen et al. (17). Mike et al. (30) opined that the surgical plane can only exist between the Toldt fascia and the deep layer of the subperitoneal fascia, which is somewhat similar to the parieto–renal interface interface mentioned by Wedel et al. (28). It is worth noting that in the Japanese anatomical literature, the subperitoneal fascia is typically used to replace the perirenal fascia. The subperitoneal fascia can be divided into two layers. The deep layer extending to the posterior abdominal wall is called the anterior renal fascia, and the superficial layer extending to the posterior abdominal wall is named the posterior renal fascia. Therefore, Mike et al. (30) considered that the separation plane lies between the Toldt fascia and the anterior renal fascia. Hohenberger et al. (3) and West et al. (37) suggested that the surgical plane was between the mesocolon and its underlying fascia (Toldt fascia). Liang et al. (27) and Garcia-Granero et al. (29) proposed that the surgical plane should be established within the Toldt fascia.

In summary, the comprehension of the surgical plane reflects the understanding of the retroperitoneal fascia system of the right hemicolon. Gastrointestinal surgeons are obliged to strengthen their learning in this domain to ensure the radical resection of tumors and minimize the occurrence of intraoperative complications.

## Discussion

Over the past century, the mesocolon has been described as fragmented and incoherent (8, 9). Nevertheless, a safe colectomy depends on the continuity of the mesocolon (38, 39). Specifically, in 2009, Hohenberger et al. (3) proposed that complete mesocolic excision (CME) significantly reduced the postoperative local recurrence rate of colon cancer and improved the 5-year survival rate. A recent systematic review also demonstrated that CME can improve the tumor prognosis of patients with right-sided colon cancer, especially those with stage III colon cancer (40). However, the fascial system and surgical plane behind the right hemicolon continue to be a controversial topic, which may be due to nomenclature confusion, inaccurate descriptions, or differences in understanding (41–43).

During embryonic development, after intestinal rotation, the right colon adheres to the parietal peritoneum of the posterior abdominal wall and the pancreaticoduodenum. The initial confusion is related to the formation of Toldt fascia. Concerning what Toldt fascia is, Mike et al. proposed that Toldt fascia belongs to fusion fascia and cannot be separated (30, 31). Interestingly, the study by Wedel et al. (28) suggested that Toldt fascia actually refers to the parietal peritoneal fascia. They hypothesized that during the adhesion of the right hemicolon, the mesothelium disappeared and was replaced by fibrous connective tissue. Consequently, the dorsal mesentery of the right hemicolon transformed into mesocolic fascia, and the parietal peritoneum of the posterior peritoneum became the parietal peritoneal fascia, namely Toldt fascia. Strictly speaking, the Toldt fascia referred to by Wedel et al. is not the same as that referred to by Mike et al. Even Carl Toldt himself did not give a clear description of the retrocolic fascia system or the “white line”. So, what exactly is the Toldt fascia? Fortunately, thanks to the pioneering study by Coffey et al. (17), we know that after intestinal rotation, even though the right hemicolon mesocolon fuses with the posterior peritoneum, the mesothelium does not vanish and remains intact until the adult stage. The loose connective tissue between the two layers of the mesothelium is the actual Toldt fascia (13, 17). The studies by Feng Bo et al. (44) and Liang et al. (27) also support this view. Although Coffey et al. did not clarify Fredet fascia, similarly, according to this theory, the classification of Fredet fascia as fusion fascia remains a subject of controversy. The study of Garcia-Granero et al. (29) also showed from the side that Fredet fascia is similar to Toldt fascia.

In right hemicolectomy, the selection of the surgical plane is of great significance, especially for elderly patients (4, 45, 46). However, it remains a controversial issue, and one of the reasons is the diverse understandings of the Toldt fascia. Coffey/Culligen et al. (17) proposed that the Toldt fascia is a layer of loose, collagen-rich connective tissue within the right hemicolon system, and the surgical plane lies either above or below the Toldt fascia. Although Wedel et al. (28) also held the view that the surgical plane should be above or below the Toldt fascia, the Toldt fascia they mentioned in their article was different from that described by Coffey et al. (17). Although the interpretation of the Toldt fascia in the study by Liang et al. (27) is consistent with that of Coffey et al. (17), they believe that the surgical plane is established within the Toldt fascia layers. Hao Huang et al. (47) suggested that in the absence of Toldt fascia invasion, the surgical plane should be above the Toldt fascia. Due to the popularization of laparoscopic and robotic surgical techniques, the surgical field can be magnified several-fold or even more than ten-fold, and the observation of the fine anatomical structures during the operation exceeds the limit of the naked-eye vision. The observation of the membrane structure under the magnified field of view seems to confirm that the Toldt fascia is a loose connective tissue gap.

Based on previous studies (17, 28, 30), it is well-established that in laparoscopic right hemicolon surgery, when mobilizing the dorsal mesocolon using the duodenum as a guide, if the duodenum is observed to have been lifted to the ventral side, away from the surface of the inferior vena cava, the surgical plane might be beneath the Toldt's fascia. Overly deep manipulation can lead to damage to the ureter and genital vessels. When approaching the descending duodenum, the pancreaticoduodenal region should be dissected following the method described by Garcia-Granero et al. (29), using the Fredet's fascia as a landmark to prevent damage to the pancreaticoduodenum.

In conclusion, the current terminology remains inconsistent. Nevertheless, the fusion-space concept seems to be the most useful in surgical practice and is histologically supported. Therefore, international standardization is urgently needed.
